# Autoimmune Pancreatitis Associated With Progressive Giant Multilocular Pancreatic Pseudocyst and Steroid-Induced Diabetes

**DOI:** 10.7759/cureus.64946

**Published:** 2024-07-19

**Authors:** Mei Yang, Zhaoqian Zhang, Sajid Zafar

**Affiliations:** 1 Internal Medicine, St. Luke's Hospital, Chesterfield, USA; 2 Gastroenterology and Hepatology, St. Luke's Hospital, Chesterfield, USA

**Keywords:** autoimmune pancreatitis (aip), panreatitis, type2 diabetes mellitus, steroid-induced diabetes, giant pancreatic pseudocyst

## Abstract

Autoimmune pancreatitis (AIP) is acknowledged as a benign ailment with swift responsivity to corticosteroid treatment (CST). Though past assumptions dismissed its connection to cyst formation, a few instances of AIP-linked pancreatic cysts (PCs) have been documented. While some cases responded positively to CST, others demonstrated resistance, necessitating intervention. Our case is a 50-year-old male with a known diagnosis of type 1 AIP. This case presents a specific adverse drug reaction of glucocorticoid that causes diabetes mellitus. Glucocorticoid was tapered due to clinical improvement and diabetes complications but also caused multiple flares. Additionally, in several months, CT showed progressive enlarging multi-cystic pancreatic head lesions, which cause constriction at the distal duodenal outlet and biliary ductal dilation. This case presents a specific adverse drug reaction of glucocorticoid that causes diabetes mellitus. Meanwhile, the fast-growing multi-cysts in the pancreatic head after treatment of type 1 AIP were very rare.

## Introduction

Autoimmune pancreatitis (AIP) is a worldwide disease and a type of chronic pancreatitis that is likely caused by immune dysregulation. Generally, AIP's overall prevalence is higher in Asia than in the Americas, Europe, or Africa, which has a very good response to glucocorticoid therapy [1,2]. There are two types of AIP. Type 1 and 2 AIP can be limited to the pancreas or present as part of other diseases. For example, type 1 AIP could involve other organs as an IgG4-related-related disease syndrome. Type 2 AIP can happen in inflammatory bowel disease (IBD) [1-4]. Formerly, the prevailing notion was that AIP did not lead to the development of chronic pancreatitis coupled with cyst formation or pancreatolithiasis [2]. Nevertheless, a few cases involving AIP-related pancreatic cysts (PCs) have been documented since the year 2003 [3].

## Case presentation

The patient is a 50-year-old male with a known diagnosis of chronic autoimmune pancreatitis confirmed by endoscopic ultrasound (EUS)-guided pancreatic biopsy and elevated IgG4 level. The patient was hospitalized multiple times for a flare-up of acute pancreatitis with severe abdominal pain, associated with nausea and vomiting. The patient was put on 40 mg of prednisone and pain management each time. One month ago, he came to his primary care physician's office with blurry vision and generalized weakness. His glucose level was in the 600s. The patient has no prior personal or family history of diabetes. He was diagnosed with steroid-induced type 2 diabetes mellitus. Metformin 1000 mg twice daily, glipizide 10 mg twice daily, and insulin aspart 100 units/mL, per sliding scale as needed when glucose larger than 150 were prescribed. However, glycemic control deteriorated while on the high dose of prednisone, which encouraged the use of taper prednisone.

**Figure 1:**
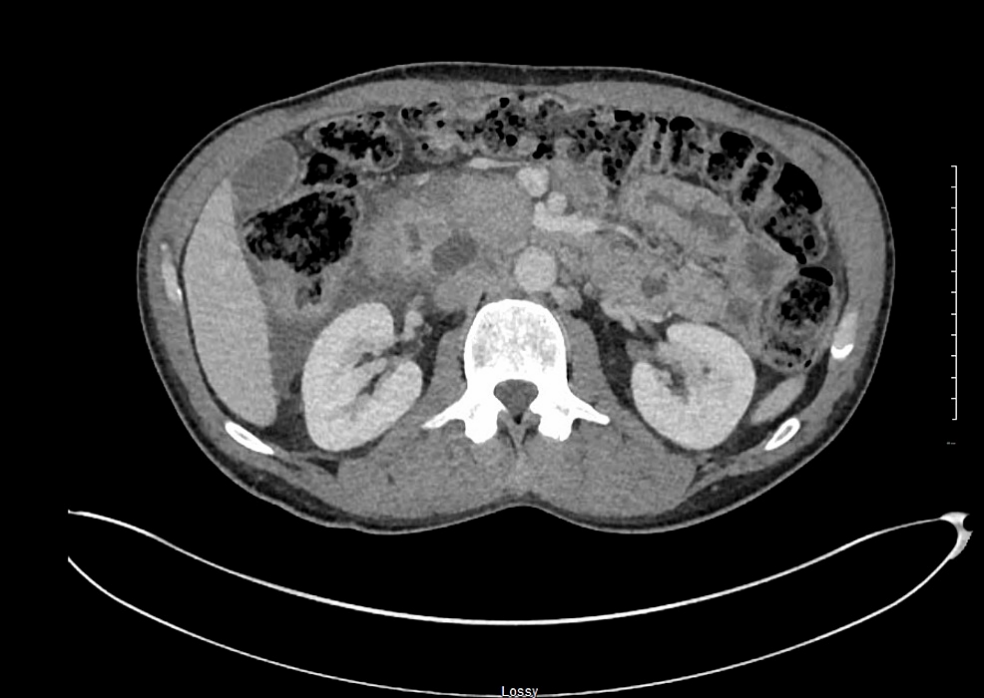
CT abdomen with contrast showed that there is s diffuse enlargement of the pancreas with the development of multiple pancreatic cysts along the pancreatic head and uncinate process measuring up to 2.4 cm

**Figure 2:**
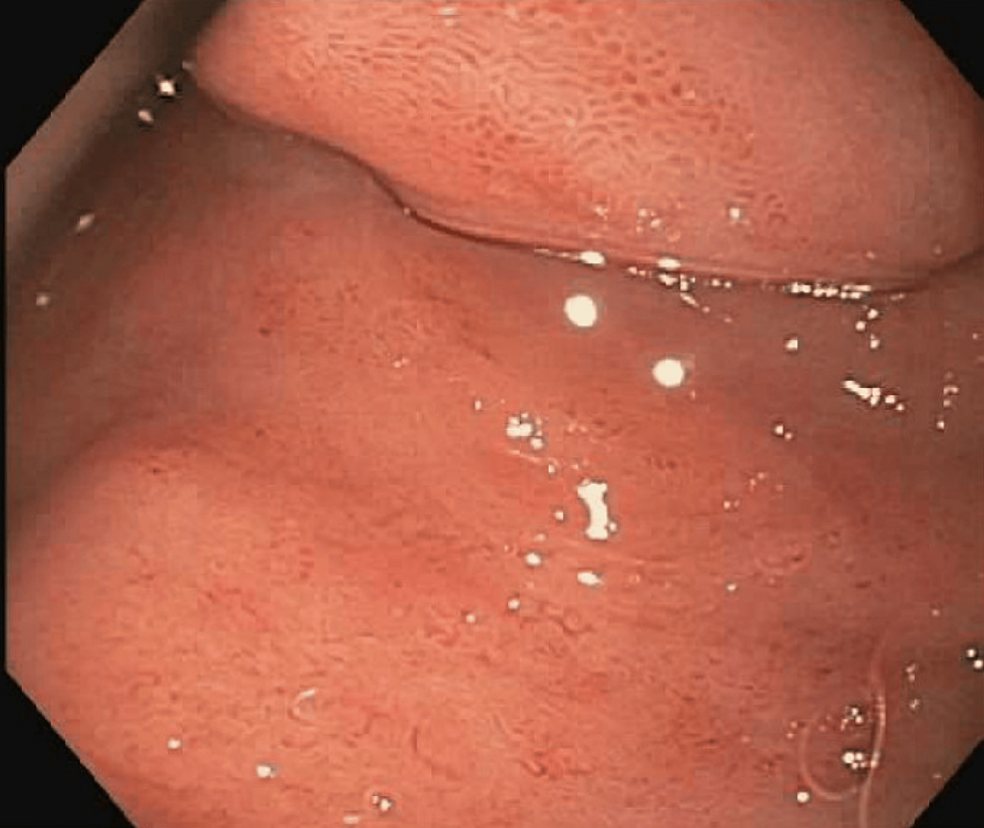
EGD showed that an acquired extrinsic severe stenosis was found in the first portion of the duodenum due to external compression

The patient was admitted to our hospital with progressive severe abdominal pain associated with nausea and vomiting for a couple of days. Vitals were stable, labs showed glucose of 174, a1c of 6.6, lipase of 1485, and lactic acid of 2.8, which trended down to 1.2. Prednisone 40 mg daily, low dose sliding scale of insulin, famotidine 40 mg daily, lactated ringer's solution 200 mL/hour, and morphine 2 mg every two hours as needed. The patient can tolerate a low-fat diet. CT abdomen with contrast showed that there is a diffuse enlargement of the pancreas with the development of multiple pancreatic cysts along the pancreatic head and uncinate process measuring up to 2.4 cm (Figure 1). Magnetic resonance cholangiopancreatography (MRCP) without contrast showed abnormal pancreatic head enlargement and multiple cystic areas in the pancreatic head consistent with a history of pancreatitis. The patient stayed two days in the hospital, and his symptoms were much better. The patient was discharged to home with his home medications and followed up with the GI clinic. One week later, the patient returned to the hospital with similar symptoms associated with nausea and vomiting and was treated with prednisone. Meanwhile, CT abdomen showed diffuse enlargement of the pancreas with worsened multiple pancreatic cysts along the pancreatic head and uncinate process measuring up to 8.5cm, with duodenal obstruction. Upper endoscopy showed an acquired extrinsic severe stenosis in the first portion of the duodenum, which continued through the duodenal sweep into the duodenum (Figure 2).

**Figure 3:**
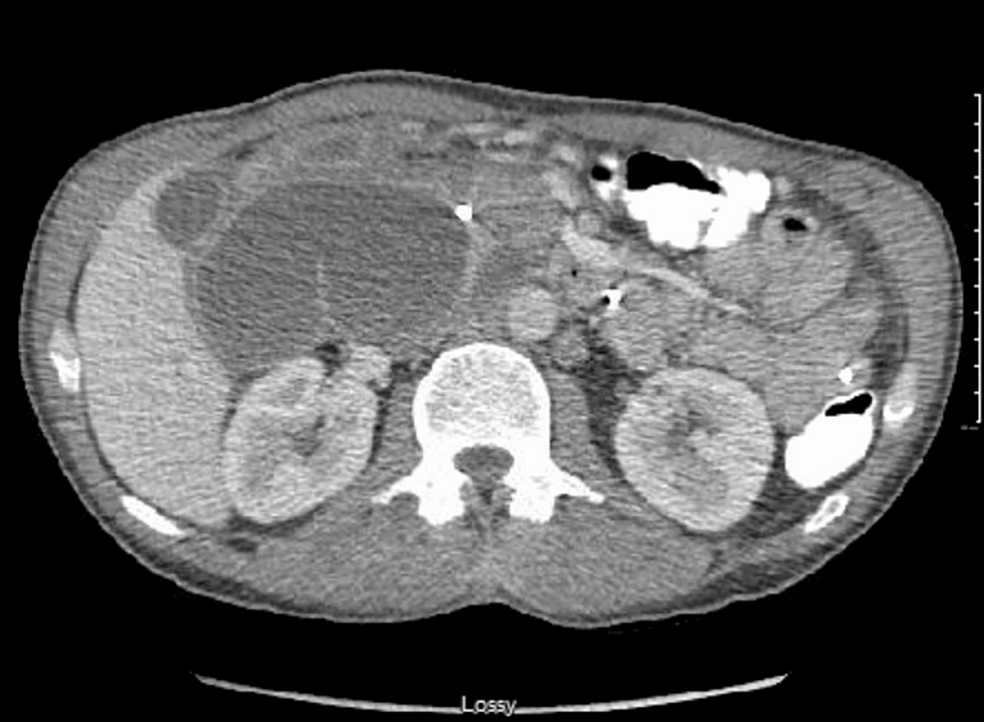
CT abdomen with contrast showed that, between the pancreatic head and gallbladder, there is a large multilocular cystic collection with an enhancing wall, which continues to increase in size, measuring at least 10.6 cm in the greatest axial dimension

Enlarging pseudocysts are an issue although they do not appear to have mature walls yet to be drained. One month later, the patient was admitted for a clogged NG tube, and a CT abdomen showed that there was a large multilocular cystic collection with an enhancing wall with a size of at least 10.6 cm in the greatest axial dimension (Figure 3). Upper endoscopy showed one non-bleeding cratered duodenal ulcer with no stigmata of bleeding, which was found in the duodenal bulb. The lesion was 8 mm in the largest dimension. An acquired benign-appearing, intrinsic moderate stenosis was found in the second portion of the duodenum and was traversed. A guide wire was inserted into the duodenum, and the endoscope was removed. A 10 Fr Naso jejunal tube was advanced over the guide wire into the duodenum. Placement was confirmed by fluoroscopy. Unfortunately, the patient still has developing fluid collections in the pancreas that are not amenable to drain due to a lack of walled-off fluid collections. Eventually, we recommend the patient follow up with GI for repeat imaging and consider endoscopic cyst gastrostomy once the wall of cysts is mature. We also recommend discharging him home on prednisone 40 mg daily for the next four weeks.

## Discussion

According to the reviewed literature, AIP was unrelated to the formation of PCs. Nevertheless, several accounts have surfaced documenting instances of AIP co-occurring with a PC [2,3]. Nonetheless, there have been occasional instances where surgical or interventional radiology interventions were chosen [4]. As of now, a unanimous agreement regarding the optimal therapeutic approach for managing PCs that coincides with AIP is lacking. Pseudocysts identified in individuals with AIP might indicate a notably intense inflammatory process; in earlier cases, it was observed that the formation of pseudocysts took place nearly two years after the commencement of abdominal symptoms [3]. Muraki et al. noted the emergence of PC pseudocyst development in AIP is a consequence of prolonged disease activity [3]. However, our case presents a rare feature in that the pseudocyst was detected within a span of one month and displayed a rapidly progressive increase in size, from 2.4 cm to 10.6 cm.

Corticosteroid treatment is clinically, radiologically, and serologically effective and has become accepted as a standard treatment for AIP. There have been some studies on the short-term effectiveness of including those receiving corticosteroid treatment [5]. It is difficult for all patients with AIP to be treated with corticosteroid, and there is currently no consensus on when to initiate corticosteroid treatment in patients with AIP. Moon et al. [6] reported that a growing pancreatic pseudocyst developed in an AIP patient who did not receive corticosteroid treatment, while Shuto et al. [7] reported the spontaneous disappearance of a PC in an AIP patient without corticosteroid treatment. Sohn et al. [8] suggested that, in AIP patients, corticosteroid treatment should be started immediately after pseudocyst appearance because of the possibility of pseudocyst regression with treatment. In our case, the patient had severe side effects of prednisone treatment, including hyperglycemia, generalized weakness, and blurry vision. The treatment approach involved endoscopic dilation and the placement of an NJ tube as symptomatic management. We are also considering subsequently performing an endoscopic cyst gastrostomy once the cyst walls have matured.

## Conclusions

Type 1 AIP manifests as a pancreatic representation of IgG4-related disease and typically exhibits positive responses to steroid therapy. Nevertheless, unusual and less frequent complications of autoimmune pancreatitis, new onset type II diabetes mellitus from steroid treatment, and multiple flares from steroid tapering happened in our case. There is a need for additional investigations into the prognosis and tailored regimen for this type of AIP. Meanwhile, the patient has a very rare steroid-resistant progressive enlarging immature pancreatic pseudocyst, which happened in one month. Endoscopic dilation and the placement of an NJ tube were provided, and later endoscopic cyst gastrostomy will be considered once the cyst walls have matured. Multiple different management methods should be thought of based on individual situations if corticosteroid treatment cannot improve the problem.
